# Data in support of a harmine-derived beta-carboline *in vitro* effects in cancer cells through protein synthesis

**DOI:** 10.1016/j.dib.2017.05.006

**Published:** 2017-05-05

**Authors:** Annelise Carvalho, Jennifer Chu, Céline Meinguet, Robert Kiss, Guy Vandenbussche, Bernard Masereel, Johan Wouters, Alexander Kornienko, Jerry Pelletier, Véronique Mathieu

**Affiliations:** a Laboratoire de Cancérologie et Toxicologie Expérimentale, Faculté de Pharmacie, Université Libre de Bruxelles, Brussels, Belgium; b Department of Biochemistry, McGill University, Montreal, Québec, Canada; c Namur Medicine and Drug Innovation Center (NAMEDIC-NARILIS), Université de Namur, Namur, Belgium; d Laboratory for the Structure and Function of Biological Membranes, Faculté des Sciences, Université Libre de Bruxelles, Brussels, Belgium; e Department of Chemistry and Biochemistry, Texas State University, 601 University Drive, San Marcos, TX 78666, USA^*^

**Keywords:** beta-carboline, protein synthesis, cancer cells

## Abstract

A harmine-derived beta-carboline, CM16, inhibits cancer cells growth through its effects on protein synthesis, as described in “A harmine-derived beta-carboline displays anti-cancer effects *in vitro* by targeting protein synthesis” (Carvalho et al., 2017)[1]. This data article provides accompanying data on CM16 cytostatic evaluation in cancer cells as well as data related to its effects on transcription and translation. After confirming the cytostatic effect of CM16, we investigated its ability to arrest the cell cycle in the glioma Hs683 and SKMEL-28 melanoma cell lines but no modification was evidenced. According to the global protein synthesis inhibition induced by CM16 [1], transcription phase, a step prior to mRNA translation, evaluated by labelled nucleotide incorporation assay was not shown to be affected under CM16 treatment in the two cell lines. By contrast, mRNA translation and particularly the initiation step were shown to be targeted by CM16 in [1]. To further decipher those effects, we established herein a list of main actors in the protein synthesis process according to literature survey for comparative analysis of cell lines displaying different sensitivity levels to CM16. Finally, one of these proteins, PERK, a kinase regulating eIF2-α phosphorylation and thereby activity, was evaluated under treatment with CM16 in a cell-free system.

## 1 Specifications table

**Table:** 

Subject area	*Biology*
More specific subject area	Protein synthesis inhibition of cancer cells *in vitro*
	Mechanism of action of potential anticancer drug
Type of data	*Graphs and table*
How data was acquired	*Flow cytometer, microplate reader, search on databases*
Data format	*Analyzed graphs and raw data retrieval (table)*
Experimental factors	*As in the description of the data and materials and methods*
Experimental features	*As in the description of the data and materials and methods*
Data source location	*Lab. de Cancérologie et Toxicologie Experimentale, Université Libre de Bruxelles, Brussels, Belgium.*
	*Life Technologies,* Madison, USA
Data accessibility	*Data is with this article*

## 2 Value of the data

• This data offers an extended comprehension of CM16 mechanism of action as a protein synthesis inhibitor in cancer cells.
• Assays performed to evaluate transcription and translation initiation provide valuable data and may be used as tools in other cell-based investigations of potential protein synthesis inhibitors.
• The data presented shows that different methods add to and enrich the investigation of the mechanism of action of proteins synthesis inhibitors in cancer cells. Therefore, these approaches might be useful in similar studies.

## 3 Data

**Fig. 1:**
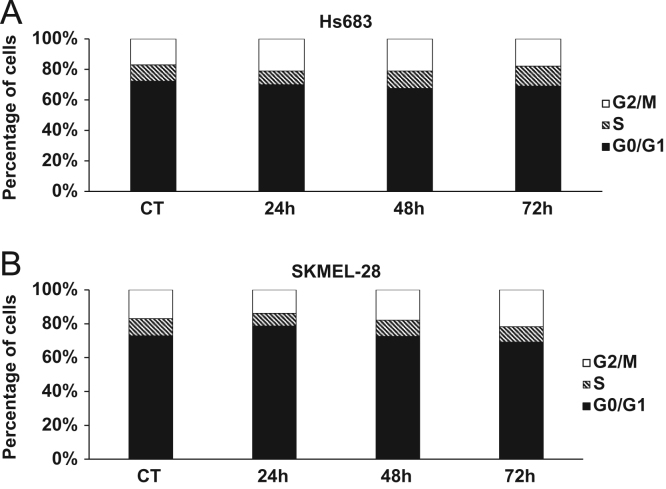
Cell cycle effects of CM16 on A: Hs683 at 0.1 µM; and B: SKMEL-28 at 0.5 µM. Data are expressed as the mean percentage of cells in each phase of the cell cycle of four replicates. As proliferation inhibition were observed on the three cancer models under study at their GI_50_ and the lack of evidence of any effects of CM16 on the cell cycle of Hs683 and SKMEL-28, we did not perform the cell cycle analysis on MDAMB-231.

**Fig. 2:**
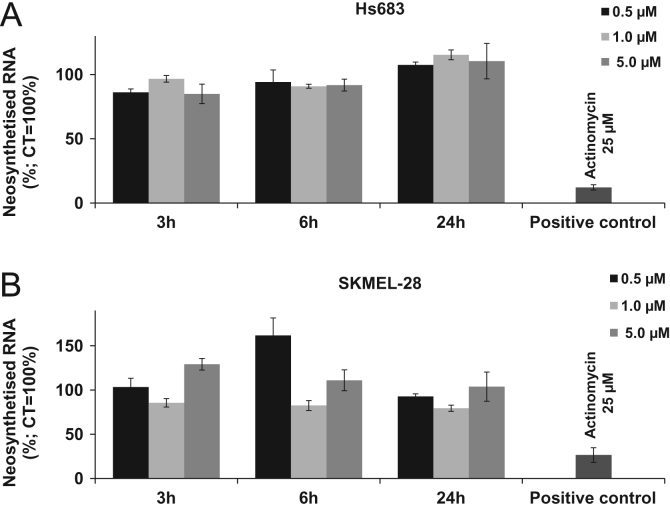
Effects of CM16 on newly synthesized mRNA in A: Hs683 and B: SKMEL-28 cell lines. Results are expressed as the mean neosynthesized RNA amounts normalized to the control (100%) ± S.E.M. of six replicates. No significant effects were observed for up to 24 h in the presence of 5.0 µM CM16 in those two cell lines, thus we did not further assayed the breast cancer cell line MDA-MB-231.

**Fig. 3:**
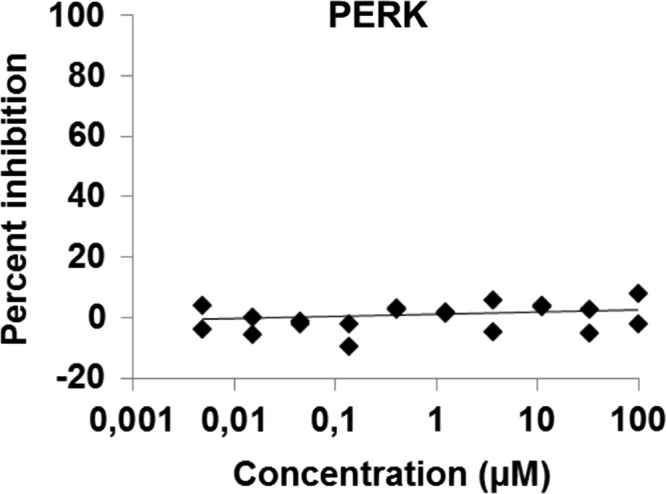
PERK kinase activity *in vitro* in the presence or absence of CM16.

Firstly, data on CM16-induced cytostatic effects is presented. As shown in [1] CM16 displays cytostatic effects at its IC_50_ in glioma Hs683, melanoma SKMEL-28 and breast adenocarcinoma MDA-MB-231 cells. Thus, CM16 effect on the cell cycle of both glioma Hs683 (Fig. 1A) cells and SKMEL-28 (Fig. 1B) are presented. After data showing CM16 inhibiting translation [1], further investigation on the effects of CM16 on newly synthesized mRNA (transcription) were carried out and generated the data here shown (Fig. 2A-B). CM16 effects on PERK activity, is shown in Fig. 3. The data on Table 1 refers to the genes related to translation that were analyzed for their transcriptomic expression in the cell lines most and least sensitive to CM16 effects, according to the NCI 60-cell-line growth inhibitory evaluation [1].

**Table 1:** List of genes analyzed for the transcript intensity from the NCI cell line panel.

Protein	Protein code (UniProt)	Gene (HGNC Symbol)	Gene code (Entrez Gene)
Eukaryotic translation initiation factor 2 subunit 1	P05198	EIF2S1	1965
Eukaryotic translation initiation factor 2 subunit 2	P20042	EIF2S2	8894
Eukaryotic translation initiation factor 2 subunit 3	P41091	EIF2S3	1968
Translation initiation factor eIF-2B subunit alpha	Q14232	EIF2B1	1967
Translation initiation factor eIF-2B subunit beta	P49770	EIF2B2	8892
Translation initiation factor eIF-2B subunit gamma	Q9NR50	EIF2B3	8891
Translation initiation factor eIF-2B subunit delta	Q9UI10	EIF2B4	8890
Translation initiation factor eIF-2B subunit epsilon	Q13144	EIF2B5	8893
Eukaryotic translation initiation factor 4E	P06730	EIF4E	1977
Eukaryotic translation initiation factor 4E-binding protein 1	Q13541	EIF4EBP1	1978
Eukaryotic translation initiation factor 4 gamma 1	Q04637	EIF4G1	1981
Eukaryotic translation initiation factor 4 gamma 2	P78344	EIF4G2	1982
Eukaryotic translation initiation factor 4 gamma 3	O43432	EIF4G3	8672
MAP kinase-interacting serine/threonine-protein kinase 1	Q9BUB5	MKNK1	8569
MAP kinase-interacting serine/threonine-protein kinase 2	Q9HBH9	MKNK2	2872
Eukaryotic initiation factor 4A-I	P60842	EIF4A1	1973
Eukaryotic initiation factor 4A-II	Q14240	EIF4A2	1974
Eukaryotic initiation factor 4A-III	P38919	EIF4A3	9775
Programmed cell death protein 4	Q53EL6	PDCD4	27250
Eukaryotic translation initiation factor 5A-1	P63241	EIF5A	1984
Eukaryotic translation initiation factor 5A-2	Q9GZV4	EIF5A2	56648
Eukaryotic translation initiation factor 5B	O60841	EIF5B	9669
Eukaryotic translation initiation factor 6	P56537	EIF6	3692
Eukaryotic translation initiation factor 1	P41567	EIF1	10209
Eukaryotic translation initiation factor 1A, X-chromosomal	P47813	EIF1AX	1964
Eukaryotic translation initiation factor 1A, Y-chromosomal	O14602	EIF1AY	9086
Probable RNA-binding protein EIF1AD	Q8N9N8	EIF1AD	84285
Eukaryotic translation initiation factor 3 subunit A	Q14152	EIF3A	8661
Eukaryotic translation initiation factor 3 subunit B	P55884	EIF3B	8662
Eukaryotic translation initiation factor 3 subunit H	O15372	EIF3H	8667
Eukaryotic translation initiation factor 3 subunit I	Q13347	EIF3I	8668
Eukaryotic translation initiation factor 3 subunit M	Q7L2H7	EIF3M	10480
Eukaryotic translation initiation factor 3 subunit E	P60228	EIF3E	3646
Eukaryotic translation initiation factor 3 subunit F	O00303	EIF3F	8665
Eukaryotic translation initiation factor 2-alpha kinase 3	Q9NZJ5	EIF2AK3	9451
Eukaryotic translation initiation factor 2-alpha kinase 4	Q9P2K8	EIF2AK4	440275
Interferon-induced, double-stranded RNA-activated protein kinase	P19525	EIF2AK2	5610
Eukaryotic translation initiation factor 2-alpha kinase 1	Q9BQI3	EIF2AK1	27102
Elongation factor 1-alpha 1	P68104	EEF1A1	1915
Elongation factor 2	P13639	EEF2	1938
Serine/threonine-protein kinase mTOR	P42345	MTOR	2475
RAC-alpha serine/threonine-protein kinase	P31749	AKT1	207
RAC-beta serine/threonine-protein kinase	P31751	AKT2	208
RAC-gamma serine/threonine-protein kinase	Q9Y243	AKT3	10000
Ribosomal protein S6 kinase beta-1	P23443	RPS6KB1	6198
Ribosomal protein S6 kinase beta-2	Q9UBS0	RPS6KB2	6199
Myc proto-oncogene protein	P01106	MYC	4609
Phosphatidylinositol 4,5-bisphosphate 3-kinase catalytic subunit alpha isoform	P42336	PIK3CA	5290
Phosphatidylinositol 3,4,5-trisphosphate 3-phosphatase and dual-specificity protein phosphatase PTEN	P60484	PTEN	5728
Hamartin	Q92574	TSC1	7248
Tuberin	P49815	TSC2	7249
Cellular tumor antigen p53	P04637	TP53	7157
Retinoblastoma-associated protein	P06400	RB1	5925
3-phosphoinositide-dependent protein kinase 1	O15530	PDPK1	5170
Mitogen-activated protein kinase 1	P28482	MAPK1	5594
Vascular endothelial growth factor A	P15692	VEGFA	7742
78 kDa glucose-regulated protein	P11021	HSPA5	3309

Data retrieved from: www.proteinatlas.com; www.uniprot.org; www.genenames.org and http://www.ncbi.nlm.nih.gov/gene in September 2015.

## 4 Experimental design, materials and methods

### 4.1 Cell lines and compound

The human cancer cell lines, oligodendroglioma Hs683 (ATCC code HTB-138) and melanoma SKMEL-28 (ATCC code HTB-72) were herein used. Cells were cultivated at 37 °C with 5% CO_2_ in RPMI culture medium supplemented with 10% FBS, 200U penicillin–streptomycin, 0.1 mg/ml gentamicin and 4 mM L-glutamine. CM16 was synthetized as previously described [2] and the experiments were designed with the cell lines described above treated with different concentrations of CM16, based on its IC_50_.

### 4.2 Analysis of CM16 effects on cell cycle

Cell cycle analysis was performed with flow cytometry through the measurements of DNA content with propidium iodide. Hs683 and SKMEL-28 were seeded in cell culture flasks and left untreated or treated with CM16 at its respective IC_50_ in each cell line for 24 h, 48 h and 72 h. The samples were then centrifuged (10 min, 1500 rcf, 4 °C), resuspended in PBS and pellets were resuspended in cold ethanol 70% for fixation. Staining with 0.08 mg/ml propidium iodide solution (0.08 mg/ml PI; 0.2 mg/ml RNAse in PBS) followed after a PBS wash. The samples were incubated at 37 °C for 30 min and stored at 4 °C for a few hours. Analysis was performed with the Cell Lab Quanta (Beckman Coulter, Analis, Suarlée, Belgium). The experiment was performed once in quadruplicate.

### 4.3 Analysis of CM16 effects on transcription

Neosynthesized RNA was evaluated through incorporation of a nucleoside analog, 5-ethynyl-uridine, using the Click iT-RNA HCS (Invitrogen, Life Technologies, Merelbeke, Belgium). The alkyne-containing nucleosides react with a fluorescent dye containing the azide moiety after their incorporation into cellular RNA. Briefly, Hs683 or SKMEL-28 cells were seeded and after attachment they were either left untreated (negative control) or treated with CM16 or the positive control actinomycin (Life Technologies, Paisley, UK). After the treatment with the analog 5-ethynyl uridine (4 mM) for two h, the cells were fixed, stained (Alexa Fluor 488 and 594) and fluorescence readings (ex/em: 495/520 nm) were carried out in microplate reader (SynergyMX Biotek, Winooski, USA: ex/em: 350/460 nm). Normalization according to cell number was carried out as described in the user manual with Hoescht counterstaining. The experiment was performed once in sextuplicate.

### 4.4 PERK inhibition

PERK activity was evaluated by the Life Technologies screening service (Lantha Screen, Madison, USA). The *in vitro* assay used is based on FRET between a terbium-labeled antibody and the phosphorylated product of the active kinase: TR-FRET increases proportionally to their binding and thereby to the quantity of the phosphorylated product. CM16 compound at different concentrations or the control solutions were mixed with the kinase/substrate/ATP mixture into the wells. After 60 min of reaction at room temperature, the detection mix was added and left to equilibrate for an h prior to fluorescence reading.

## References

[1] Carvalho A., Chu J., Meinguet C., Kiss R., Vandenbussche G., Masereel B. (2017). A harmine-derived beta-carboline displays anti-cancer effects in vitro by targeting protein synthesis. Eur. J. Pharmacol..

[2] Meinguet C., Bruyère C., Frédérick R., Mathieu V., Vancraeynest C., Pochet L. (2015). 3D-QSAR, design, synthesis and characterization of trisubstituted harmine derivatives with in vitro antiproliferative properties. Eur. J. Med. Chem..

