# Supplementary material for: Data in support of a harmine-derived beta-carboline in vitro effects in cancer cells through protein synthesis
Source: Data Brief. 2017 May 5;12:546–51. doi: 10.1016/j.dib.2017.05.006 (PMC5429240; doi:10.1016/j.dib.2017.05.006)

AUTHOR DECLARATION

Manuscript :

Data in support of a harmine-derived beta-carboline in vitro effects in cancer cells through protein synthesis

Conflict of interest : none

The manuscript contains an acknowledgement section with all the detailed financial supports of the present study. Accordingly, we wish to confirm that there are no known conflicts of interest associated with this publication and there has been no significant financial support for this work that could have influenced its outcome.

On behalf of all co-authors,

The corresponding author : Véronique Mathieu

Date : 2nd May 2017

Signature :

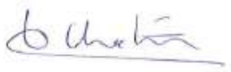

Supplement: Supplementary file 1 — Supplementary material [file mmc1.pdf]
